# Antioxidant Effect of Melatonin in Preterm Newborns

**DOI:** 10.1155/2021/6308255

**Published:** 2021-11-19

**Authors:** Lucia Marseglia, Eloisa Gitto, Elisa Laschi, Maurizio Giordano, Carmelo Romeo, Laura Cannavò, Anna Laura Toni, Giuseppe Buonocore, Serafina Perrone

**Affiliations:** ^1^Department of Human Pathology of the Adult and Developmental Age, University of Messina, Messina, Italy; ^2^Department of Molecular and Developmental Medicine, University of Siena, Siena, Italy; ^3^Department of Clinical Medicine and Surgery, Federico II University, Naples, Italy; ^4^Department of Medicine and Surgery, University of Parma, Parma, Italy

## Abstract

**Introduction:**

Preterm infants are at risk of free radical-mediated diseases from oxidative stress (OS) injury. Increased free radical generation has been demonstrated in preterm infants during the first seven days of life. Melatonin (MEL) is a powerful antioxidant and scavenger of free radicals. In preterm neonates, melatonin deficiency has been reported. Exogenous melatonin administration appears a promising strategy in the treatment of neonatal morbidities in which OS has a leading role.

**Objective:**

The aim was to evaluate plasma MEL concentrations and OS biomarkers in preterm newborns after early administration of melatonin.

**Methods:**

A prospective, randomized double-blind placebo-controlled pilot study was conducted from January 2019 to September 2020. Thirty-six preterm newborns were enrolled. Starting from the first day of life, 21 received a single dose of oral melatonin 0.5 mg/kg once a day, in the morning (MEL group); 15 newborns received an equivalent dose of placebo (placebo group). Samples of 0.2 mL of plasma were collected at 24 and 48 hours after MEL administration. Plasma concentrations of melatonin, non-protein-bound iron (NPBI), advanced oxidation protein products (AOPP), and F2-isoprostanes (F2-Isopr) were measured. Babies were clinically followed until discharge.

**Results:**

At 24 and 48 hours after MEL administration, the MEL concentrations were significantly higher in the MEL group than in the placebo group (52759.30 ± 63529.09 vs. 28.57 ± 46.24 pg/mL and 279397.6 ± 516344.2 vs. 38.50 ± 44.01 pg/mL, respectively). NPBI and AOPP did not show any statistically significant differences between the groups both at 24 and 48 hours. At 48 hours, the mean blood concentrations of F2-Isopr were significantly lower in the MEL group than in the placebo group (36.48 ± 33.85 pg/mL *vs.*89.97 ± 52.01 pg/mL).

**Conclusions:**

Early melatonin administration in preterm newborns reduces lipid peroxidation in the first days of life showing a potential role to protect high-risk newborns. *Trial Registration*. This trial is registered with NCT04785183, Early Supplementation of Melatonin in Preterm Newborns: the Effects on Oxidative Stress.

## 1. Introduction

Preterm infants are at risk for neonatal disorders related to immaturity. A common factor in the pathogenesis of such diseases is the free radical-mediated tissue injury derived from oxidative stress (OS) [1]. The endogenous indoleamine melatonin, synthesized from the neurotransmitter serotonin, is a powerful broad-spectrum antioxidant and readily available scavenger of free radicals. Foetal melatonin has a maternal origin, and after birth, the full-term neonates have an irregular melatonin secretion for 3-5 months, leading to a transient melatonin deficiency in the neonatal period and in the first months of life. Prematurity delays the maturation of the neurological network that controls melatonin secretion, leading to poor secretion for an even longer period. Furthermore, the onset of pineal melatonin secretion seems to be even more delayed in case of neurological damage, and this event, together with other predisposing conditions, makes the preterm even more susceptible to the free radical-mediated damage [2–4]. Therefore, exogenous melatonin administration appears a promising strategy in the treatment of neonatal morbidities in which OS has a leading role. Moreover, as it shows neuroprotective properties, it was present as a joint therapy in addition to hypothermia after hypoxic-ischemic encephalopathy [5–8]. Several studies tested the efficacy of melatonin to counteract oxidative damage in diseases of newborns such as chronic lung disease, perinatal brain injury, necrotizing enterocolitis, retinopathy of prematurity, and sepsis, giving promising results [9–11]. In these studies, the dosages of melatonin varied over a wide range, ranging from 0.1 to 100 mg/kg. This is an evidence that the pharmacokinetic profile of melatonin is better known in adults than newborns [12]. Indeed, just few studies investigated pharmacokinetic characteristics of melatonin in preterm and asphyxiated neonates. Merchant et al. observed and described a decreased volume of distribution and prolonged half-life and clearance of the melatonin in preterm infants with respect to adults and older children. Melatonin was administered intravenously at the dosage of 0.1 mg/kg for two hours [13]. Carloni et al. investigated the melatonin pharmacokinetics at comparable doses after intragastric administration in human preterm infants. The main result of the study was that a single intragastric bolus of 0.5 mg/kg of melatonin resulted in higher serum melatonin level than adults suggesting the possibility to get and keep therapeutic concentrations with this dose [14]. Finally, Balduini et al. demonstrated that a safe and potentially effective dose of melatonin for infants with hypoxic ischemic encephalopathy undergoing hypothermia should not exceed 5 mg/kg, depending on the route of administration [15]. However, no data are available on the therapeutic efficacy of these specific doses. The aim of this study was to evaluate melatonin concentrations and biomarkers of oxidative stress in preterm infants after early administration of melatonin.

## 2. Materials and Methods

This prospective randomized double-blind placebo-controlled pilot study was conducted at the Neonatology Unit of the Polyclinic in Messina from January 2019 to September 2020. The study was approved by local Ethical Committee (protocol number 42/2018). Written informed consent was obtained from parents. Inclusion criteria were gestational age < 37 weeks and normal liver and kidney function tests. Exclusion criteria are all outborn babies, babies with severe congenital malformations, sepsis, inborn errors of metabolism, suffering from perinatal hypoxia, or born from mothers with mental disorders, to eliminate conditions that could affect melatonin production. Additional exclusion criteria were as follows: withdraw informed consent, insufficient blood sample, and hemolysis of the blood sample. The MEL group received an oral dose of 0.5 mg/kg of melatonin, once a day in the morning, in the first week of life; the placebo group received 0.5 mL of 5% glucose solution. Newborns received melatonin (Pisolino® Gocce, Pediatrica, Italy) by a nasogastric tube. Pisolino® Gocce contains fructose, purified water, potassium sorbate, sodium benzoate, flavorings, and xanthan gum. The product is present in the register of food supplements of the Ministry of Health website (http://www.ministerosalute.it/alimenti/dietetica) and classified with the following code: 62853.

This product is subject to the European Directive on foods according to the DL n. 169 of 21/05/2004 and not to the European Directive on medicines 2001/20/EC implemented at Italian level with D.L. n. 211 of 06/24/2003. Melatonin administration has a good safety profile, with no known adverse effects [16]. Plasma concentrations of non-protein-bound iron (NPBI) (micromol/L), advanced oxidation protein products (AOPP) (micromol/dL), and F2-isoprostanes (F2-Isopr) (pg/mL) were determined at 24 and at 48 hours after administration of melatonin. The primary endpoint was to evaluate MEL concentration in the MEL group and placebo group. The secondary endpoint was to evaluate biomarkers of OS, such as AOPP, NPBI, and F2-Isopr in the MEL and placebo groups. Further, the occurrence of intraventricular hemorrhage (IVH), necrotizing enterocolitis (NEC), retinopathy of prematurity (ROP), and bronchopulmonary dysplasia (BPD) in all enrolled preterm newborns was analysed.

### 2.1. Procedures

Blood samples (0.5 mL) were collected, by vein puncture, from each newborns at 24 and 48 hours after administration of MEL. The samples were immediately centrifuged (RTM 1500, *T* 4°C, 10 min) to remove cells and obtain the supernatant, which was then separated into two different microtest tubes, one of which contained BHT (butylated hydroxytoluene), and stored at −80°C. The obtained samples were subsequently analysed to measure melatonin and OS biomarker (AOPP, F2-IsoPs, and NPBI) concentrations. Plasma melatonin concentrations were measured by high-performance liquid chromatography and mass spectrometry (MS/MS) (Agilent Technologies 1200 series system and an AB Sciex API 4000 triple-quadrupole mass spectrometer) according to the method of Wang et al. [17]. Markers of protein and lipid peroxidation were measured by AOPP and F2-Isopr. Spectrophotometry, tandem mass spectrometer coupled with HPLC, and HPLC-DAD system were used to analyse AOPP, F2-Isopr, and NPBI [18–20]. AOPP were measured using spectrophotometry on a microplate reader. The instruments were calibrated with chloramine-T solutions that absorb at 340 nm in the presence of potassium iodide [18]. The LC-MS/MS method of Casetta et al. [19] was followed for determination of F2-IsoPs. The method was centered around an API 4000 tandem mass spectrometer coupled with HPLC Agilent 1200 series, which includes a binary pump, a thermostated well-plate autosampler, and a column over. Chromatography separation was carried out at a temperature of 30°C by a mixture of an aqueous solution of acetic acid (Eluent 1) and acetonitrile (Eluent 2). For measurements, the tandem mass spectrometer ran in multiple reaction monitoring with the electroscopy source operating in negative ion mode and by exploiting the transitions *m*/*z*353.3 > 193.2 for F2 IsoPr and 357.3 > 197.2 for the internal standard d4-8-iso-PGF_2*α*_. The method of Paffetti et al. [20] was followed for NPBI measurement with HPLC-DAD system (Agilent 1100 series). The method is based on preferential chelation of NPBI by a large excess of the low-affinity ligand disodium nitrilotriacetic acid. To separate NPBI, a two-step filtration procedure was used: (1) filtration through a 100 kDa Vecta-Spin Micro-Whatman ultracentrifuge filter and (2) filtration through a 20 kDa Vecta Spin Micro-Whatman ultracentrifuge filter at 13.660 × g and 4°C. The filtrate was injected directly into an isocratic reverse-phase liquid chromatography system using precolumn derivatization with the high-affinity iron ligand DHP, which forms a coloured complex with Fe3+ that absorbs at 450 nm. The analytic system detected iron as a ferric nitrate standard down to a concentration of 0.01 *μ*M.

### 2.2. Statistical Analysis

A computer-generated randomization schedule was used to define the supplemented group (MEL group) or control (placebo group). Due to lacking data on oral melatonin supplementation in preterm newborns, sample size was calculated by G∗Power 3.9.1.2 for windows [21], estimating that between the 2 groups, there was a large difference in the concentration of melatonin (setting: effect size: 0.8, alfa error: 0.5, and power: 0.80); the minimum sample size required was 28. Statistical analysis was performed by SPSS version 25.0 for Windows (IBM, Armonk, NY, USA). Normal distribution of data was evaluated by Kolmogorov-Smirnov test. Data with nonnormal distribution and categorical data were evaluated by Mann–Whitney *U* test and chi-square test, respectively. A *p* value < 0.05 was considered statistically significant.

## 3. Results

**Figure 1:**
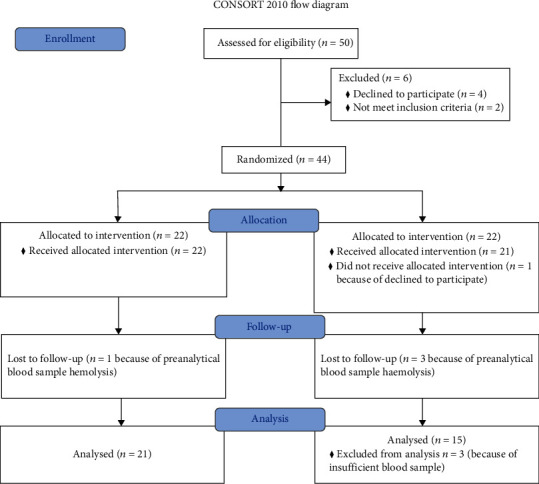
Consort diagram 2010.

**Figure 2:**
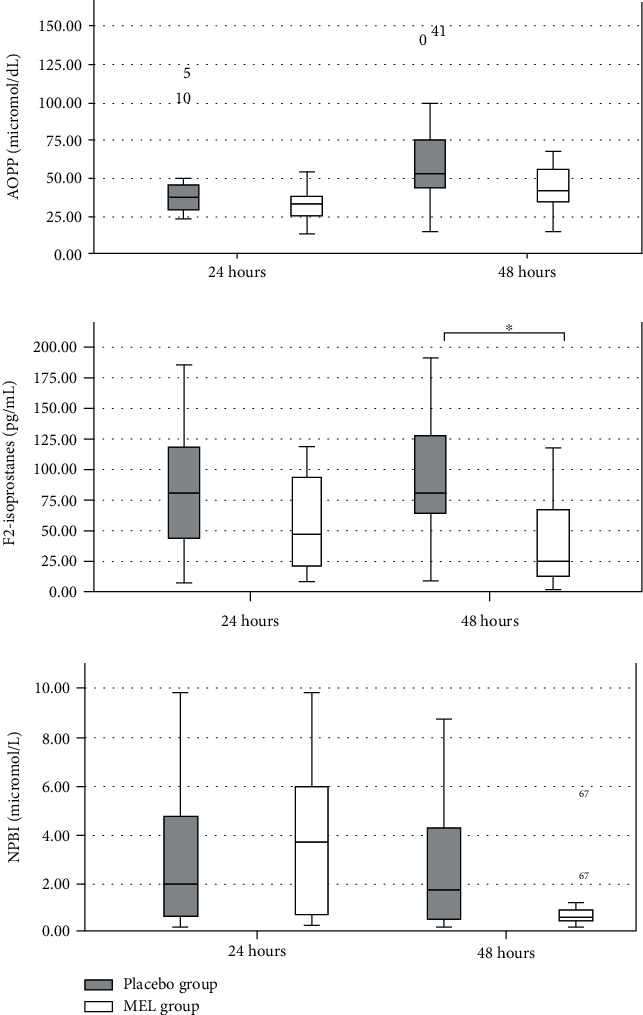
Isoprostanes, NPBI, and AOPP concentrations in placebo and MEL groups at 24 and 48 hours after melatonin administration. ^∗^*p* < 0.05. Data are expressed as median (Q1-Q3). AOPP: advanced oxidative protein products; NPBI: non-protein-bound iron.

The flow diagram of the study population from assessment for eligibility to analysis is reported in Figure 1. Out of the 36 consecutively enrolled preterm newborns, 21 received melatonin (MEL group) and 15 received placebo (placebo group).  Table 1 reports baseline characteristics of the enrolled population. Melatonin concentrations were significantly higher in the MEL group at 24 and 48 hours (Table 2). In the placebo group, male showed significantly higher concentrations of melatonin than female at 24 hours of life (58.1 ± 55.4*vs.*2.8 ± 3.5; *p* = 0.001); in the MEL group, female showed significantly higher concentrations of melatonin concentration than male at 48 hours of life (302296.3 ± 372402.9*vs.*22781.0 ± 35155.7; *p* = 0.03). No statistical difference between groups were found in AOPP and NPBI at 24 and 48 hours; also, F2-Isopr was not different at 24 hours (Table 2). At 48 hours, the mean plasma concentrations of F2-Isopr were significantly lower in the MEL group than in the placebo group (36.48 ± 33.85*vs.*89.97 ± 52.01 pg/mL, *p* < 0.05; Table 2, Figure 2). No differences between male and female in OS biomarkers were observed.

## 4. Discussion

The inability to counteract the harmful effects of free radicals is a matter of concern for all newborns, especially if preterm. The transition from intrauterine to extrauterine environment is characterized by a huge of oxygen availability [11, 22]. This new hyperoxic condition increases the generation of various reactive oxygen species (ROS) such as hydrogen peroxide, singlet oxygen, and hydroxyl radicals that may attack macromolecules and cellular components. Moreover, ROS, as a secondary messenger, may trigger signalling pathways and induce stress-response genes or proteins [22, 23]. A significant increase in total hydroperoxides and AOPP levels from birth to 7 days of life has been reported in preterm newborns, indicating that damage caused from free radicals also occurs in nonhypoxic babies with normal clinical course [24]. Experimental studies in an animal model of hypoxic-ischemic brain damage report the effectiveness of antioxidant drugs to prevent or reduce ROS injury. Melatonin has been demonstrated to be able to block OS and inflammation pathways [25, 26]. In the first days of life, numerous factors could be responsible for an overproduction of free radicals, such as hypoxia, hyperoxia, acidosis, infections, transfusions, drug exposure, and pain [27]. Newborns are therefore peculiarly at high risk for OS-induced damage [28]. Therefore, there is compelling evidence that supplementation with antioxidant compounds may be effective in combating OS. Melatonin has not only free radical scavenging and antioxidant properties but also anti-inflammatory, antiapoptotic, and analgesic actions. Indeed, melatonin seems to modulate both pro- and anti-inflammatory cytokines in various pathophysiological situations wherein the balance between them determines the clinical outcome and to inhibit the expression of cyclooxygenase and inducible nitric oxide synthase, the nitric oxide production induced by lipopolysaccharide, and the inflammasome activation [11]. This fact is of clinical importance if we consider that inflammation is strictly related to OS in the pathogenesis of many diseases that affect preterm newborns [1]. Previous reports have suggested that preterm infants do not secrete melatonin until 52-week postconception [29]. In our study, we were able to measure the melatonin concentration in plasma of preterm infants who received placebo. All subjects received maternal or human donor milk which was a potential source of exogenous melatonin, being present in human milk [30]. Melatonin concentrations were found significantly higher in male than female in the placebo group at 24 hours of life and in female than male in the MEL group at 48 hours of life. To our knowledge, no data on melatonin differences between male and female have been reported. Immature hepatic metabolism and poor renal excretion may be responsible for a wider range of melatonin concentrations in treated preterm babies. Whatever the reason for the observed gender differences, the data should be checked in a large population due to the variability of melatonin concentrations in preterm newborn.

A protective effect of melatonin on lipoperoxidation was observed when orally administered in preterm newborns in the first days of life. Significantly lower levels of F2-Isopr were found in the MEL than the placebo group at 48 hours of life. This result is particularly important since early measurement of F2-Isopr has been recently described to discriminate patients showing abnormal white matter injury score at term of corrected gestational age with a cutoff value 31.8 pg/mL [31]. Moreover, high levels of urinary F2-Isopr were found in second days of life in newborns at high risk of developing a hemodynamically significant patent ductus arteriosus [32]. Increased levels of F2-Isopr have been also reported in preterm newborns affected by bronchopulmonary dysplasia or periventricular leukomalacia [33]. It was demonstrated that F2-Isopr provokes preoligodendrocyte death by oncosis, depending on inadequate antioxidant defences [34]. White matter injury, bronchopulmonary dysplasia, periventricular leukomalacia, and patent ductus arteriosus represent some of the peculiar diseases of prematurity, now grouped and called “free radical diseases of prematurity” because of the common pathways in pathogenesis [1]. The results of the present pilot prospective study show that few doses of melatonin decrease lipid peroxidation in preterm supplemented newborns. Thus, melatonin appears to reduce the risk of oxidative damage, protecting vulnerable organs and tissues in preterm newborns. F2-Isopr are the *in vivo* result of free radical-induced injury by peroxidation of lipids in cell membranes. They are stable compounds generated by the action of cyclooxygenase on long-chain unsaturated fatty acids. The mechanism involved in their formation implies that free radicals cause hydrogen abstraction from arachidonic acid and addition of molecular oxygen to form a peroxyl radical. F2-Isopr are terminal oxidation products with no further oxidant properties, therefore representing reliable markers of OS in newborns [35]. AOPP are the terminal products of the protein exposure to free radicals without oxidant properties, and they represent a marker of the degree of protein damage in oxidative stress conditions. We previously reported an increase of AOPP levels from birth to seventh day of life in preterm newborns [24]; in this paper, we observed a lower relative increment of AOPP level in treated newborns than controls. NPBI is a low-molecular-mass iron form, free from binding to plasma proteins. Iron toxicity derives from the production of hydroxyl radicals through the Fenton reaction. Thus, NPBI is a marker of potential OS because it indicates increased susceptibility to oxidative damage especially in vivo studies [35]. We previously found an association between NPBI and lipid oxidation in vitro [36]. In this study, no significant effect on NPBI and AOPP was observed at 24 and 48 hours from MEL administration, plausibly due to the small sample size associated with wide variability in biomarker plasma concentrations. Data could be also probably related to the multifactorial nature of the oxidative stress processes and to the need of higher doses of melatonin than those used. Furthermore, no significant effects were found on the prevalence of NEC, BPD, IVH, and ROP in the MEL group than the placebo group. It is noteworthy that the population study represented preterm newborns at medium-low risk to develop these diseases (mean gestational age > 28 weeks in both groups). Melatonin supplementation in extremely low birth weight or gestational age infants might have a major potentiality to reduce the increase of lipoprotein oxidation products. To our knowledge, lack of data exists regarding the valuation of melatonin efficacy in reducing term and preterm infant morbidity. This study has the limitation of few patients enrolled, and the results need to be confirmed in larger trials. However, the results reported support for the first time the role of melatonin intake to protect preterm newborns against lipid peroxidation. The potential protective role of MEL is mainly due to its beneficial effect on plasma antioxidant status. Moreover, the safety profile of melatonin in clinical study is an encouraging start point for further investigate the protective effects of melatonin on organs and tissues. Our results pave the way for more medical research in this field before melatonin enters in clinical practice. Further research is needed as the schedule that might be effective and the subjects that might receive melatonin to obtain the greatest effect have not been precisely defined.

## Figures and Tables

**Table 1:** Clinical characteristics of enrolled population.

	MEL group (*n* = 21)	Placebo group (*n* = 15)	*p* value
Gestational age (wks)	32.26 ± 3.66	33.53 ± 2.88	NS
Birth weight (g)	1706 ± 638	1988 ± 513	NS
Gender (%)	*F* = 14 (66)	*F* = 8 (53)	NS
Spontaneous delivery (%)	5 (24)	2 (13)	NS
Caesarean section (%)	16 (76)	13 (87)	NS
NEC (%)	1 (4.7)	0	NS
BPD (%)	1 (4.7)	0	NS
IVH (all grade) (%)	4 (19)	3 (20)	NS

F: female; NEC: necrotizing enterocolitis; BPD: bronchopulmonary dysplasia; IVH: intraventricular hemorrhage; ROP: retinopathy of premature; NS: nonsignificant *p* > 0.05.

**Table 2:** Melatonin, AOPP, NPBI, and F2-Isopr levels in test and control groups at 24 and at 48 hours of life.

	24 hours			48 hours		
	Placebo group	MEL group	*p* value	Placebo group	MEL group	*p* value
	Mean ± SD [median (25°-75°)]			Mean ± SD [median (25°-75°)]		
Melatonin (pg/mL)	28.57 ± 46.24 [10 (1-43)]	52759.30 ± 63529.09 [18309 (8886-100831)]	<0.001^∗^	38.50 ± 44.01 [17 (4-121)]	279397.6 ± 516344.2 [37349 (10108-274844)]	<0.001^∗^
NPBI (micromol/L)	2.40 ± 3.46 [0.7 (0.2-3.3)]	3.97 ± 3.13 [4 (1-6)]	0.113	2.99 ± 3.56 [0.8 (0.1-6)]	2.23 ± 2.37 [1 (0.6-5)]	0.525
AOPP (micromol/dL)	44.66 ± 26.54 [36 (28-45)]	36.07 ± 16.03 [32 (24-42)]	0.297	54.96 ± 24.33 [53 (33-75)]	51.66 ± 18.11 [47 (38-60)]	0.715
F2-Isoprostanes (pg/mL)	82.47 ± 51.30 [80 (31-121)]	75.05 ± 87.75 [46 (20-93)]	0.168	89.97 ± 52.01 [80 (62-127)]	36.48 ± 33.85 [24 (10-68)]	<0.008^∗^

Data are expressed as mean ± SD and median (25-75°C).

## Data Availability

The data that support the findings of this study are available on request from the corresponding author.

## References

[1] Perrone S., Santacroce A., Longini M., Proietti F., Bazzini F., Buonocore G. (2018). The free radical diseases of prematurity: from cellular mechanisms to bedside. *Oxidative Medicine and Cellular Longevity*.

[2] Muñoz-Hoyos A., Bonillo-Perales A., Avila-Villegas R. (2007). Melatonin levels during the first week of life and their relation with the antioxidant response in the perinatal period. *Neonatology*.

[3] Kennaway D. J., Goble F. C., Stamp G. E. (1996). Factors influencing the development of melatonin rhythmicity in humans. *The Journal of Clinical Endocrinology & Metabolism*.

[4] Biran V., Decobert F., Bednarek N. (2019). Melatonin levels in preterm and term infants and their mothers. *International Journal of Molecular Sciences*.

[5] Balduini W., Carloni S., Perrone S. (2012). The use of melatonin in hypoxic-ischemic brain damage: an experimental study. *The Journal of Maternal-Fetal & Neonatal Medicine*.

[6] El Farargy M. S., Soliman N. A. (2020). A randomized controlled trial on the use of magnesium sulfate and melatonin in neonatal hypoxic ischemic encephalopathy. *Journal of Neonatal-Perinatal Medicine*.

[7] Cardinali D. P. (2019). An assessment of melatonin's therapeutic value in the hypoxic-ischemic encephalopathy of the newborn. *Frontiers in Synaptic Neuroscience*.

[8] Hobson A., Baines J., Weiss M. D. (2013). Beyond hypothermia: alternative therapies for hypoxic ischemic encephalopathy. *The Open Pharmacology Journal*.

[9] Xu Y., Lu X., Hu Y. (2018). Melatonin attenuated retinal neovascularization and neuroglial dysfunction by inhibition of HIF-1*α*-VEGF pathway in oxygen-induced retinopathy mice. *Journal of Pineal Research*.

[10] Zhang W. X., He B. M., Wu Y., Qiao J. F., Peng Z. Y. (2019). Melatonin protects against sepsis-induced cardiac dysfunction by regulating apoptosis and autophagy via activation of SIRT1 in mice. *Life Sciences*.

[11] Tarocco A., Caroccia N., Morciano G. (2019). Melatonin as a master regulator of cell death and inflammation: molecular mechanisms and clinical implications for newborn care. *Cell Death & Disease*.

[12] Andersen L. P., Werner M. U., Rosenkilde M. M. (2016). Pharmacokinetics of oral and intravenous melatonin in healthy volunteers. *BMC Pharmacology and Toxicology*.

[13] Merchant N. M., Azzopardi D. V., Hawwa A. F. (2013). Pharmacokinetics of melatonin in preterm infants. *British Journal of Clinical Pharmacology*.

[14] Carloni S., Proietti F., Rocchi M. (2017). Melatonin pharmacokinetics following oral administration in preterm neonates. *Molecules*.

[15] Balduini W., Weiss M. D., Carloni S. (2019). Melatonin pharmacokinetics and dose extrapolation after enteral infusion in neonates subjected to hypothermia. *Journal of Pineal Research*.

[16] Andersen L. P., Gögenur I., Rosenberg J., Reiter R. J. (2016). The safety of melatonin in humans. *Clinical Drug Investigation*.

[17] Wang A. Q., Wei B. P., Zhang Y., Wang Y. J., Xu L., Lan K. (2011). An ultra-high sensitive bioanalytical method for plasma melatonin by liquid chromatography-tandem mass spectrometry using water as calibration matrix. *Journal of Chromatography B, Analytical Technologies in the Biomedical and Life Sciences*.

[18] Witko-Sarsat V., Friedlander M., Capeillère-Blandin C. (1996). Advanced oxidation protein products as a novel marker of oxidative stress in uremia. *Kidney International*.

[19] Casetta B., Longini M., Proietti F., Perrone S., Buonocore G. (2012). Development of a fast and simple LC-MS/MS method for measuring the F2-isoprostanes in newborns. *The Journal of Maternal-Fetal & Neonatal Medicine*.

[20] Paffetti P., Perrone S., Longini M. (2006). Non-protein-bound iron detection in small samples of biological fluids and tissues. *Biological Trace Element Research*.

[21] Faul F., Erdfelder E., Lang A. G., Buchner A. (2007). G∗Power 3: a flexible statistical power analysis program for the social, behavioral, and biomedical sciences. *Behavior Research Methods*.

[22] Perez M., Robbins M. E., Revhaug C., Saugstad O. D. (2019). Oxygen radical disease in the newborn, revisited: oxidative stress and disease in the newborn period. *Free Radical Biology and Medicine*.

[23] Tipple T. E., Ambalavanan N. (2019). Oxygen toxicity in the neonate: thinking beyond the balance. *Clinics in Perinatology*.

[24] Buonocore G., Perrone S., Longini M. (2002). Oxidative stress in preterm neonates at birth and on the seventh day of life. *Pediatric Research*.

[25] Hardeland R. (2018). Melatonin and inflammation-story of a double-edged blade. *Journal of Pineal Research*.

[26] Wang Z., Zhou F., Dou Y. (2018). Melatonin alleviates intracerebral hemorrhage-induced secondary brain injury in rats via suppressing apoptosis, inflammation, oxidative stress, DNA damage, and mitochondria injury. *Translational Stroke Research*.

[27] Di Fiore J. M., Vento M. (2019). Intermittent hypoxemia and oxidative stress in preterm infants. *Respiratory Physiology & Neurobiology*.

[28] Peña-Bautista C., Durand T., Vigor C., Oger C., Galano J. M., Cháfer-Pericás C. (2019). Non-invasive assessment of oxidative stress in preterm infants. *Free Radical Biology and Medicine*.

[29] Kennaway D. J., Stamp G. E., Goble F. C. (1992). Development of melatonin production in infants and the impact of prematurity. *The Journal of Clinical Endocrinology and Metabolism*.

[30] Illnerova H., Buresova M., Presl J. (1993). Melatonin rhythm in human milk. *The Journal of Clinical Endocrinology and Metabolism*.

[31] Coviello C., Perrone S., Buonocore G. (2021). Isoprostanes as biomarker for white matter injury in extremely preterm infants. *Frontiers in Pediatrics*.

[32] Coviello C., Tataranno M. L., Corsini I. (2020). Isoprostanes as biomarker for patent ductus arteriosus in preterm infants. *Frontiers in Pediatrics*.

[33] Ahola T., Fellman V., Kjellmer I., Raivio K. O., Lapatto R. (2004). Plasma 8-isoprostane is increased in preterm infants who develop bronchopulmonary dysplasia or periventricular leukomalacia. *Pediatric Research*.

[34] Brault S., Martinez-Bermudez A. K., Roberts J. (2004). Cytotoxicity of the E_2_-isoprostane 15-E_2t_-IsoP on oligodendrocyte progenitors. *Free Radical Biology and Medicine*.

[35] Longini M., Belvisi E., Proietti F., Bazzini F., Buonocore G., Perrone S. (2017). Oxidative stress biomarkers: establishment of reference values for isoprostanes, AOPP, and NPBI in cord blood. *Mediators of Inflammation*.

[36] Signorini C., Perrone S., Sgherri C. (2008). Plasma esterified F_2_-isoprostanes and oxidative stress in newborns: role of nonprotein-bound iron. *Pediatric Research*.

